# Robotic Distal Pancreatectomy and Splenectomy for an Intrapancreatic Hepatocellular Carcinoma: A Case Report and Review of the Literature

**DOI:** 10.1089/pancan.2020.0009

**Published:** 2020-10-27

**Authors:** Charles C. Vining, Phillip J. Hsu, Darryl Schuitevoerder, Nora E. Joseph, Melissa E. Hogg

**Affiliations:** ^1^ Department of Surgery, University of Chicago, Chicago, Illinois, USA.; ^2^ Medical Scientist Training Program, University of Chicago, Chicago, Illinois, USA.; ^3^ Department of Pathology and NorthShore University HealthSystem, Evanston, Illinois, USA.; ^4^ Department of Surgery, NorthShore University HealthSystem, Evanston, Illinois, USA.

**Keywords:** robotic, pancreatectomy, hepatocellular carcinoma

## Abstract

**Background:** Liver parenchyma that resides outside of the normal hepatic confines is defined as accessory liver if in communication with the native biliary tree, or ectopic liver (EL) if it is not. EL can develop in a variety of tissues, including but not limited to the gallbladder, the hepatic ligaments, the pancreas, and retroperitoneum. EL has an increased propensity for malignant degeneration resulting in hepatocellular carcinoma (HCC).

**Presentation:** A 67-year-old Korean male presented with epigastric discomfort and was found to have an elevation in his transaminases. Cross-sectional imaging demonstrated a 1.3 cm solid mass in the body of the pancreas with features concerning for either a pancreatic ductal adenocarcinoma or pancreatic neuroendocrine tumor. Subsequent endoscopic ultrasound and fine needle aspiration demonstrated cells of epithelial origin with hepatocellular differentiation. A robotic-assisted distal pancreatectomy and splenectomy was performed with final pathology demonstrating a well-differentiated HCC.

**Conclusions:** EL with malignant degeneration resulting in HCC requires surgical excision. The majority of patients reported with EL resulting in HCC in the pancreas have had the tumors located in the body and tail. Therefore, definitive treatment requires distal pancreatectomy and splenectomy. Herein, we describe the presentation, workup, and definitive treatment of HCC arising in the pancreas.

## Introduction

The majority of incidentally discovered solid pancreatic lesions include pancreatic ductal adenocarcinoma (PDAC), pancreatic neuroendocrine tumors (pNETs), solid pseudopapillary tumors, metastatic deposits, and focal chronic pancreatitis.^1^ However, other rare solid pancreatic lesions exist and have clinical significance.

The liver, gallbladder, and biliary ductal system development begins in the 4th week of gestation from the ventral and caudal hepatic diverticulum of the foregut endoderm during organogenesis. In proximity, the ventral and dorsal pancreatic buds develop giving rise to the pancreatic head/uncinate and neck/body/tail, respectively.^2^ Owing to inappropriate tissue migration or altered cellular differentiation, ectopic liver (EL) parenchyma can develop in various locations.^3^

Hepatocellular carcinoma (HCC) is the third most common cause of cancer-related death worldwide and most typically originates in the setting of cirrhosis secondary to hepatitis, alcohol, autoimmune hepatitis, toxin exposure, nonalcoholic steatohepatitis and metabolic diseases such as Wilson's disease and hemochromatosis.^4^ EL has an increased propensity for malignant degeneration compared with the normal liver and is frequently unrelated to cirrhosis, viral hepatitis, or other known risk factors for HCC.^5,6^

Herein, we report a case of a patient who presented with indigestion and epigastric discomfort, found to have a distal pancreatic mass, treated with a robotic distal pancreatectomy and splenectomy, with final pathology demonstrating HCC.

## Methodology

This report is exempt from institutional review board. Signed written consent was obtained from the patient to use details from the medical record without identifying the patient by name.

## Case Report

**FIG. 1.**
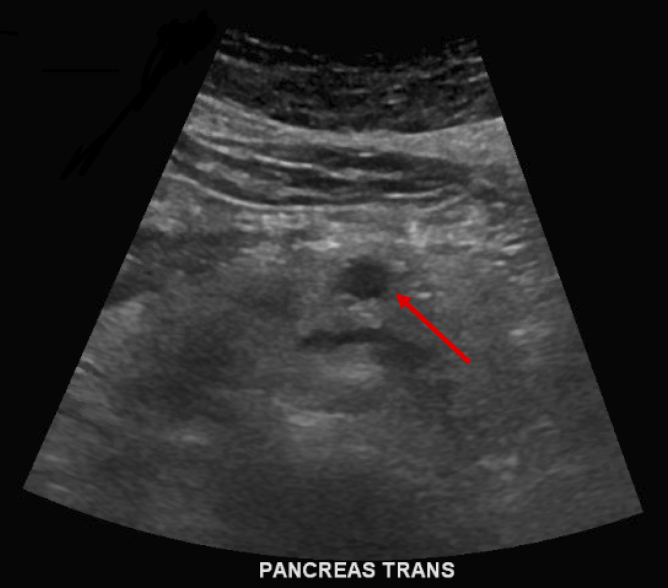
Transabdominal ultrasound demonstrating a well-circumscribed hypoechoic pancreatic lesion without pancreatic ductal dilation. Red arrow denotes the lesion.

**FIG. 2.**
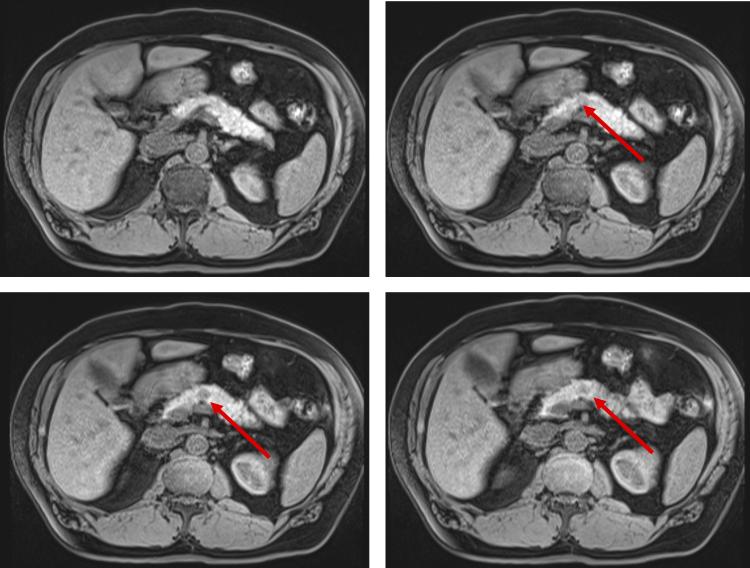
Magnetic resonance imaging of the pancreatic lesion. Red arrows denote the pancreatic lesion.

**FIG. 3.**
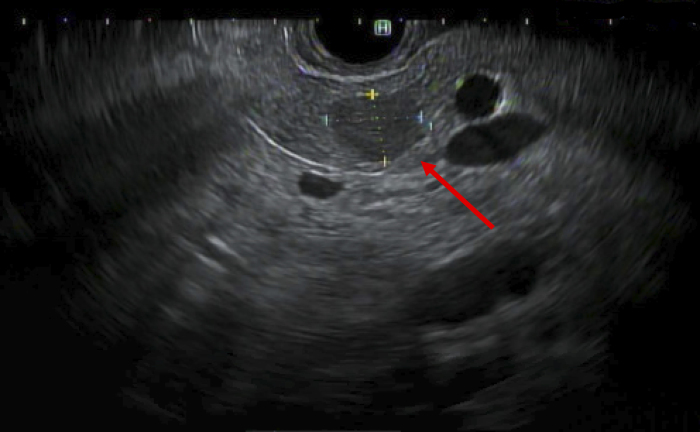
Endoscopic ultrasound demonstrating a well-circumscribed hypoechoic pancreatic lesion without pancreatic ductal dilation. Red arrow denotes the pancreatic lesion.

A 67-year-old Korean male with no past medical or surgical history presented to his primary care physician with indigestion and epigastric discomfort. His physical examination was unremarkable. Laboratory assessment demonstrated a mild elevation in his hepatic transaminases (serum glutamic oxaloacetic transaminase 43, serum glutamic pyruvic transaminase 75), which prompted a transabdominal ultrasound. A hypoechoic 1.1 cm cystic mass without significant vascularity was identified in the body of the pancreas without ductal dilation (Fig. 1). A subsequent magnetic resonance imaging (MRI) of the abdomen demonstrated a 1.3 cm indeterminate solid mass in the pancreatic body with very subtle T2 hyperintensity and a very subtle hyperintense signal on diffusion weighted images. The lesion was isointense with the background pancreatic parenchyma on postcontrast images and was suspicious for a pNET or PDAC (Fig. 2). Of note, a background of hepatic steatosis was noted. An endoscopic ultrasound with fine needle aspiration (FNA) was performed and demonstrated a hypoechoic 11.3 mm by 8.3 mm oval mass in the pancreatic body with well-defined borders (Fig. 3). Cytology from the FNA demonstrated monomorphic epithelial cells (pankeratin and cytokeratin antibody positive in a number of carcinomas [OSCAR] positivity) of hepatocellular origin (hepatocyte-specific antigen positivity) on immunohistochemistry. The Ki-67 was very low to absent. These findings were consistent with a well-differentiated HCC, concerning for either metastatic or primary HCC of the pancreas. A hepatitis panel and tumor markers such as alpha-fetoprotein (AFP), carcinoembryonic antigen, and cancer antigen 19-9 were all negative.

The case was discussed in a multidisciplinary tumor board and surgical excision was recommended. Diagnostic laparoscopy demonstrated no evidence of masses in the liver and no evidence of peritoneal or omental metastasis. A total of six ports were placed and the robot was docked. An intraoperative ultrasound demonstrated that the mass was distal to the pancreatic neck. A tunnel under the neck of the pancreas at the superior mesenteric vein and portal vein confluence was established. A stapler was used to divide the pancreas immediately medial to the gastroduodenal artery. The splenic artery and vein were dissected circumferentially and divided using a linear stapler. The remaining attachments to the retroperitoneum were divided and the specimen was removed. A drain was left adjacent to the pancreatic staple line. The patient's postoperative course was unremarkable, his drain amylase level was low, and his drain was removed before discharge on postoperative day 4.

**FIG. 4.**
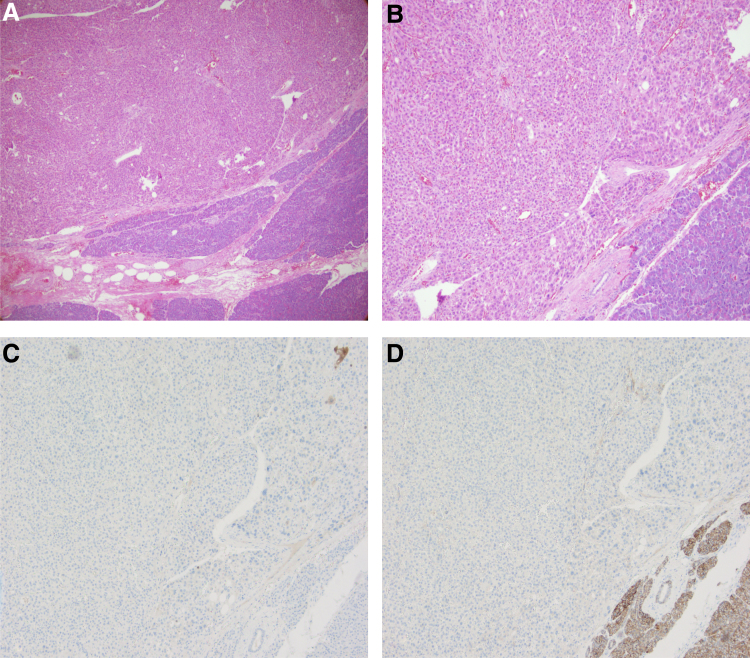
Pathology of tumor and surrounding normal pancreas. **(A)** H&E low power view of tumor (upper half) and normal pancreas (lower half). **(B)** H&E higher power (20 × ) view of the neoplastic cell population with a rim of normal pancreas, lower right-hand corner. **(C, D)** Immunohistochemical stains of the same field at higher power, stained with hepatocyte marker Arginase 1 **(C)** and epithelial marker MOC-31 **(D)**. H&E, hematoxylin and eosin; MOC-31, a monoclonal antibody against epithelial glycoprotein 2.

The final gross pathology demonstrated a 1.5 cm tan-brown solid soft well-circumscribed lesion. Rare cells contained cytoplasmic pigment suggestive of bile. The final pathology was consistent with a 1.5 cm well-differentiated carcinoma with hepatoid features (Fig. 4). All margins were negative and all 18 lymph nodes evaluated were negative for carcinoma. In addition, two incidental neuroendocrine microadenomas were identified.

## Discussion

Herein, we report a case of a patient with HCC in the distal pancreas, arising from EL, who underwent definitive treatment with robotic distal pancreatectomy and splenectomy. This case highlights the workup and treatment of a rare pancreatic lesion.

Extrahepatic tissue outside of the normal liver confines but in communication with the biliary system is defined as accessory liver, whereas extrahepatic liver tissue not in communication with the biliary system is known as EL.^7^ The most common location for EL is the gallbladder and can range in size from millimeters to several centimeters,^8,9^ whereas EL located in the pancreas is more uncommon.^10–14^ The exact incidence of EL is unknown but thought to be ∼0.24% to 0.47% (Refs.^7,8,15,16^).

EL harbors an increased risk of malignant transformation, with reported rates of ∼12% to 30% (Refs.^5,17–19^). This increased risk is likely due to the lack of native vasculature and inability of bile excursion. The functional impairment of the liver may cause chronic inflammation as well as prolonged exposure to carcinogenic factors due to inadequate removal.^5,6,20^ In addition, most patients do not have cirrhosis or the standard risk factors for HCC development. It should be noted, however, that although our patient did not have cirrhosis, the MRI did detect evidence of steatohepatitis, a risk factor for cirrhosis and subsequent HCC.

The appearance of HCC on MRI is well described and can be distinguished between a regenerative and dysplastic nodule. In the native liver, HCC demonstrates variable signal on T1-weighted precontrast images with both arterial enhancement and delayed washout. On T2-weighted images, the lesion can demonstrate an elevated signal or be isointense relative to the native liver.^21^ The radiological appearance of HCC in EL is less well characterized with less apparent arterial enhancement.^5,10,22^ The present lesion demonstrated very subtle T2 hyperintensity and a very subtle hyperintense signal on diffusion weighted images that can be characteristic of HCC; however, other diagnoses such as pNET remained plausible.

AFP is a protein produced by the fetal liver and functions in a similar manner to albumin. After birth, the level of this protein rapidly decreases; however, in the setting of hepatic regeneration and HCC, AFP production is re-established. Therefore, AFP is used as a tumor marker for the diagnosis of HCC and has prognostic significance.^23,24^ AFP was not elevated in this case, likely due to the small size and well differentiation of the lesion. This is consistent with other reports of HCC arising in EL in the literature (Table 1). Interestingly, the fucosylated form of serum AFP, AFP-L3, which is more specific for HCC, was measured in four studies in the literature, and was elevated in all four.^19,25–27^

**Table 1.** Case Reports of Primary Pancreatic Hepatocellular Carcinomas

Study	Title	Presentation	Pancreatic location	Tumor marker elevation	Risk factors	Management
Adachi et al.^19^	Ectopic hepatocellular carcinoma mimicking a retroperitoneal tumor: a case report	Incidentally found	Head	AFP (30.1 ng/mL); AFP-L3 (83.1%)	Hepatitis C antibody positive	Extirpation
Braun et al.^10^	Pure hepatocellular carcinoma originates from an ectopic liver nodule located in the pancreas	Abdominal discomfort	Tail	No	None	Distal pancreatectomy
Li et al.^11^	Multiple ectopic hepatocellular carcinomas in the pancreas: a case report	Abdominal mass and elevated AFP	Multiple: body and head	AFP (>1200 ng/mL)	Hepatitis B core antibody positive	Enucleation
Steen et al.^12^	Primary hepatocellular carcinoma (“hepatoid” carcinoma) of the pancreas: a case report and review of the literature	Incidentally found	Tail	CA 19-9 (46 U/mL) Neuron-specific enolase (16.7 μg/L)	None	Distal pancreatectomy and splenectomy
Kubota et al.^13^	Ectopic hepatocellular carcinoma arising from pancreas: a case report and review of the literature	Incidentally found	Tail	No	None	Distal pancreatectomy and splenectomy
Cardona et al.^14^	Hepatocellular carcinoma arising from ectopic liver tissue in the pancreas	Back and flank pain	Body	No	Smoking	Distal pancreatectomy and splenectomy

AFP, alpha-fetoprotein; CA, cancer antigen.

Definitive treatment of these lesions includes surgical excision and prognosis is excellent.^5,14,20^ Therefore, management of these lesions should mirror that of small localized HCC in the native liver without cirrhosis followed by surveillance.^23^ Considering the majority of pancreatic lesions are located in the distal pancreas, a minimally invasive distal pancreatectomy and splenectomy should be performed.^28^ The use of Sorafenib or Lenvatinib for HCC in EL has not been evaluated in cases such as ours, although it has demonstrated improved overall survival and increased time to radiological progression in advanced HCC located in the normal anatomic liver parenchyma.^29,30^ Considering the increased predilection for malignant degeneration in EL, routine excision of identified EL should be considered.

Primary HCC arising from EL can occur in various locations. When EL gives rise to HCC, definitive treatment is surgical excision if technically feasible. The role for adjuvant therapy is poorly defined; however, surgery, with margin negative resection, provides excellent prognosis. Further studies are required to identify risk factors for the development of HCC in EL and define risk factors for metastasis.

## Authors' Contributions

All authors have approved the final article.

## Statement of Originality

This article is original work and has not been published before and is not being considered for publication elsewhere in its final form, in either printer or electronic media.

## Abbreviations Used

AFP: alpha-fetoprotein
EL: ectopic liver
FNA: fine needle aspiration
H&E: hematoxylin and eosin
HCC: hepatocellular carcinoma
MRI: magnetic resonance imaging
PDAC: pancreatic ductal adenocarcinoma
pNETs: pancreatic neuroendocrine tumors
